# Purification of Bioactive Lipopeptides Produced by *Bacillus subtilis* Strain BIA

**DOI:** 10.1007/s10337-016-3164-3

**Published:** 2016-09-17

**Authors:** Muaaz Alajlani, Abid Shiekh, Shahida Hasnain, Adelheid Brantner

**Affiliations:** 1 Department of Pharmacognosy, Institute of Pharmaceutical Sciences, University of Graz, Universitaetsplatz 4/I, 8010 Graz, Austria; 2 Department of Microbiology and Molecular Genetics, Faculty of Life Sciences, University of the Punjab, Lahore, Pakistan; 3 Department of Microbiology and Molecular Genetics, Faculty of Life Sciences, The Women University Multan, Multan, Pakistan

**Keywords:** Purification, Lipopeptides, TLC, Gel-filtration, RP-HPLC, MALDI–TOF-MS

## Abstract

*Bacillus subtilis* strain BIA was used for the production of bioactive lipopeptides. Different extraction and purification methods were assayed as liquid–liquid extraction, and acid and ammonium sulfate precipitation followed by TLC, SPE, and gel filtration. Active fractions were further purified using RP-HPLC. The molecular mass of the purified product from HPLC was determined through Tris-Tricine SDS-PAGE and MALDI–TOF-MS. The results revealed that *Bacillus subtilis* strain BIA produced surfactin and iturin like compounds. Coproduction of surfactin and iturin like compounds by this strain is a remarkable trait for a potential biocontrol agent. This paper also includeds techniques that have been developed for the optimal and convenient extraction of bioactive lipopeptides from microbial origin.

## Introduction

Several hundred wild-type *Bacillus* spp. have the potential to produce more than two dozen antibiotics with an amazing variety of structures [1]. Non-ribosomally generated amphipathic lipopeptide antibiotics with condensed β-hydroxyl or β-amino fatty acids are widespread in *B. subtilis* [2]. These are synthesized by large multienzyme systems that have a modular structure [3]. Lipopeptide antibiotics identified so far have been divided into three main groups according to their structure: surfactin group, iturin group, and fengycin group [4]. All these agents occur as families of closely related isoforms which differ in length and branching of the fatty acid side chains and in amino-acid substitutions in the peptide rings [5]. These agents are natural compounds with a high potential for biotechnological and pharmaceutical applications [6]. They are distinguished by excellent surface and membrane-active properties along with superior emulsifying and foaming properties [7], which can be utilized in food biotechnology and in the agricultural sector. Apart from this, bioactive lipopeptides act as antifungal agents [8], antiviral agents, antiameobocytic agents, and antimycoplasma agents [9]. Surfactin is well qualified to maintain virus and mycoplasma safety in biotechnological products [10]. Their mechanism of action is ongoing and has revealed even intercellular mechanisms [11]. Lipopeptides are often extracted from culture broth by classical methods, including acidic precipitation (HCl), recrystallization, and extraction by organic solvents. Unfortunately, impurities are coextracted and the extraction must be completed by the chromatographic procedures [12]. Therefore, to purify and concentrate lipopeptides, several authors have attempted one-step methods [13], including a two-phase extraction [14], an ultrafiltration method [15], and a solid-phase extraction [16]. The technique of foam fractionation is also of great interest, as it offers the double advantage of continuous in situ removal of surfactin from the fermentation broth and prevention of any possible feed-back inhibition [17]. A *Bacillus* strain, designated as BIA exhibiting antibacterial activity, was isolated from Pakistan with spectrum of activity mostly against Gram-positive bacteria. The strain was identified on the basis of 16S rRNA and biochemical characterization. Lipopeptide analysis was performed by Tris-Tricine SDS-PAGE and matrix-assisted laser desorption ionization–time of flight-mass spectrometry (MALDI–TOF-MS). The results from Tris-Tricine SDS-PAGE showed that *B. subtilis* BIA produces an antibacterial compound with molecular mass of about 3 kDa. The exact molecular mass was determined through MALDI–TOF-MS which is effective for peptides and proteins with molecular masses ranging from 0.5 to 30 kDa. Here, we report the co-production of surfactin and iturin like compounds from *B. subtilis* BIA based on their molecular masses through MALDI–TOF-MS.

## Materials and Methods

### Bacterial Strains and Culture Conditions

Twenty-four strains isolated from different soil samples were tested for their antibiotic production using the agar-well diffusion assay [18]. One strain was selected based upon the size and clarity of zone of inhibition. The test organisms are a collection of laboratory strains as well as environmental isolates. *Bacillus fusiformis* was routinely used for the sensitivity tests. All strains were routinely maintained on nutrient agar [19], however, for antibiotic production Landy medium [20] was used.

### Antibiotic Assay

Samples of culture supernatant containing the antibiotic were assayed for activity using an agar-well diffusion assay [18]. Fifty microliter of *Bacillus fusiformis* liquid culture of 0.3 OD_600_ was spread onto the surface of Petri dish-containing l-agar. 50 μL antibiotic sample was transferred into the well made in media plates using a sterile cork borer. The sample was allowed to diffuse into the agar and the plate was inverted and incubated at 37 °C until a lawn of the indicator bacteria appeared on the plate (approximately 10–16 h).

### DNA Extraction PCR and Ribotyping

Genomic DNA was extracted from overnight incubated bacterial culture in Luria–Bertani LB-broth [19] at 37 °C with 120 rpm. The extraction was carried out using gene extraction kit (Biorad, UK). PCR amplification of 16S rDNA was performed successfully following the method described by [21] with forward primer 27f (5-AGAGTTTGATCCTGGCTCAG) and reverse primer 1522r (5-AAGGAGGTGATCCA(AG)CCGCA) [22]. To 0.5–0.1 ng of chromosomal template DNA, 0.25 μM each primer, 200 μM deoxynucleoside triphosphate, and 1 unit of *Taq* polymerase were added. Solution was heated to 94 °C for 5 min and passed through 29 cycles as follows: denaturation for 20 s at 94 °C, primer annealation for 20 s at 50 °C, and extension at 72 °C for 2 min. Final extension was at 72 °C for 5 min. The product was purified using Aqua pure extraction kit (Fermantas, UK) and sequenced using both 27f and 1522r primers by automatic sequencer (Applied Biosystems, USA).

### Solvent Extraction and Thin-Layer Chromatography Analysis

Bacterial supernatant, recovered by 15,000×*g* centrifugation for 20 min of 36-h old shaken culture, was screened for the presence of active compounds. Supernatants were extracted with same amount ethyl acetate (Fishers, USA) and vacuum dried. Residues were dissolved in minimal amount of ethyl acetate and subjected to thin-layer chromatography (TLC) sheet (Merck, Germany) and developed with 1:1:1 v/v/v of *n*-hexane, chloroform, and methanol as mobile phase. The spots were detected under UV light; by spraying with water for the detection of hydrophilic compounds; with ninhydrin for detection of compound with free amino groups [23, 24]. However, active fractions were detected using a narrow strip of developed sheets by a bioassay method with a sensitive test organism [13]. The active fraction was scrapped from the TLC plate and extracted with eluent A (0.1 % (vol/vol) trifluoroacetic acid and 20 % (vol/vol) acetonitrile).

### Acid Precipitation and Solid-Phase Extraction

Lipopeptides (LPS) were precipitated with 12-N HCl (pH 2.0) and the precipitate was extracted with minimal amount of methanol. The sample was dried and dissolved in 20 % (vol/vol) acetonitrile. This sample was fractionated through solid-phase extraction (SPE) columns (CHROMABOND C18ec) purchased from Macherey–Nagel, Germany. First columns were preconditioned with methanol then water and samples were applied under low pressure. The columns were washed with water and seven fractions were eluted by consecutive seven solutions 5, 15, 25, 35, 50, 75, and 100 % acetonitrile. The active fractions were monitored through the spot-plate method using test organism.

### Ammonium Sulfate Precipitation (ASP) and Gel Filtration Chromatography

Ammonium sulfate (Sigma) was gently added to the cell-free supernatant (maintained at 4 °C) to obtain 80 % saturation, and stirred for 4 h. After centrifugation (1 h at 20,000×*g*, 4 °C), the pellet was resuspended in 50-mM Tris–HCl, pH 7.5, and loaded on Econo-Pac 10DG column (Biorad, UK) equilibrated in 50 mM Tris–HCl, pH 7.5. The lipopeptides were eluted from the column using a flow rate of 0.5 mL/min and fractions were collected every 1 mL. The similar fractions evaluated by TLC were combined and tested for their antimicrobial activity by agar-well diffusion assay.

### Purification by Reverse Phase HPLC

Active fractions from TLC, SPE, and gel filtration were further purified by reversed-phase HPLC using Thermo Hypersil-Keystone ODS (particle size, 5 μm; column dimensions, 250 by 4.6 mm, Thermo Hypersil, USA). The sample was applied along with eluent A (Milli-Q HPLC grad water) and eluted with segmented gradients of eluent B (0.1 % vol/vol trifluoroacetic acid and acetonitrile). The system used was 40 % eluent B for 30 min and 40–100 % eluent B for 10 min.

### Tris-Tricine SDS-PAGE

Gel electrophoresis was performed according to the method of Schagger [25]. Tris-Tricine SDS-PAGE gels were made and run using a Mini Protean II slab gel electrophoresis unit (Biorad). One millimeter spacers were used to set the thickness of the gels. Each gel consisted of two portions: a 2-cm stacking gel, and a 5.0-cm separating gel. Culture supernatant samples were lyophilized and dissolved in loading buffer prior to PAGE. The purified antibiotic was dissolved directly in loading buffer. Gels were run at 40 V until the tracking dye passed stacking gel, and then, a constant voltage of 140 V was applied until the dye reached the bottom of the gel. Gels were visualized by staining with coomassie brilliant blue. Antibiotic activity was specifically located on the gel using a direct activity assay [26].

### MALDI–TOF-Mass Spectrometry Analysis

Purified compound from RP-HPLC was analyzed through matrix assistant laser desorption ionization time of flight-mass spectroscopy (MALDI–TOF-MS) to find the exact molecular mass. 2 µL of sample mixed with 2-µL matrix solution (2 mg of alpha-cyano-4-hydroxycinnaminic acid per ml in acetonitrile-methanol–water (1:1:1) on the target plate). MALDI–TOF-MS spectra were recorded using a 337-nm nitrogen laser for desorption and ionization. The mass spectrometer was operated in the linear mode at an accelerating voltage of 18 kV with an ion flight path that was 0.7-m long. The delay time was 375 ns. Matrix suppression was also used, and the mass spectra were averaged over 50–100 individual laser shots. The laser intensity was set just above the threshold for ion production. External calibration was performed using the [M + H]^+^ signals of renin, adrenocorticotropic hormone, insulin oxidized B, and bovine insulin (Sigma-Aldrich Co.).

## Results

### 16S rDNA Sequence Analysis and Phylogeny

*Bacillus subtilis* strain BIA was identified on the basis of the 16S rDNA sequence analysis. For that purpose, nearly complete 16S rDNA sequence was determined and analyzed using NCBI Website. Strain BIA showed more than 97 % homology with *B. subtilis* strain B-FS01 (DQ520955).

### Thin-Layer Chromatography

Eight prominent spots were visible under UV having *R*_*f*_ values of 0.08, 0.12, 0.21, 0.37, 0.49, 0.53, and 0.57. However, only one active fraction was observed upon bioassay with *R*_*f*_ value of 0.49. The spot was ninhydrin negative, indicating the absence of free amino groups and presence of peptide bonds in the compound. A white spot formed with same *R*_*f*_ value when the plate was sprayed with water, indicating that the compound is lipophilic. In addition, spots with *R*_*f*_ values of 0.08 and 0.53 could not be stained with ninhydrin, while all other spots were ninhydrin positive.

### Solid-Phase Extraction

**Fig. 1:**
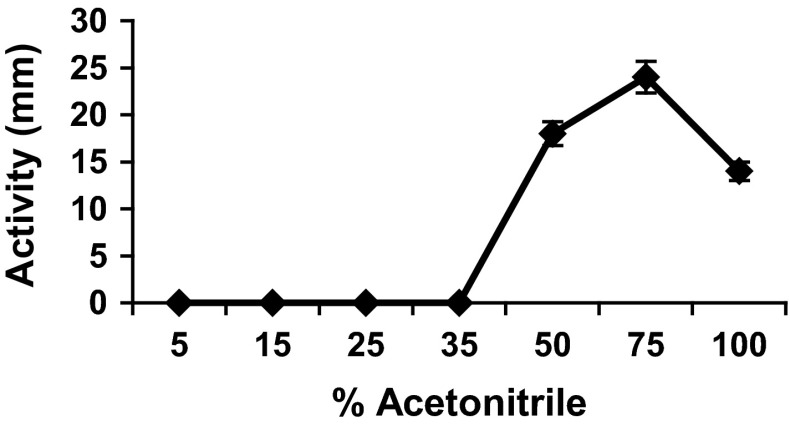
Solid-phase extraction from the acid precipitation of culture supernatant. Seven fractions were eluted with decreasing polarity and activity observed using sensitive test organism

Seven fractions were collected by decreasing the polarity with acetonitrile until 100 % and tested for activity. Fractions eluted with 5, 15, 25, and 35 % acetonitrile did not show any activity. However, three active fractions were eluted with 50, 75, and 100 % acetonitrile with peak activity at 75 % (Fig. 1), showing that maximum product is eluted when the polarity is decreased until 3/4 to that of start.

### Gel Filtration Chromatography

**Fig. 2:**
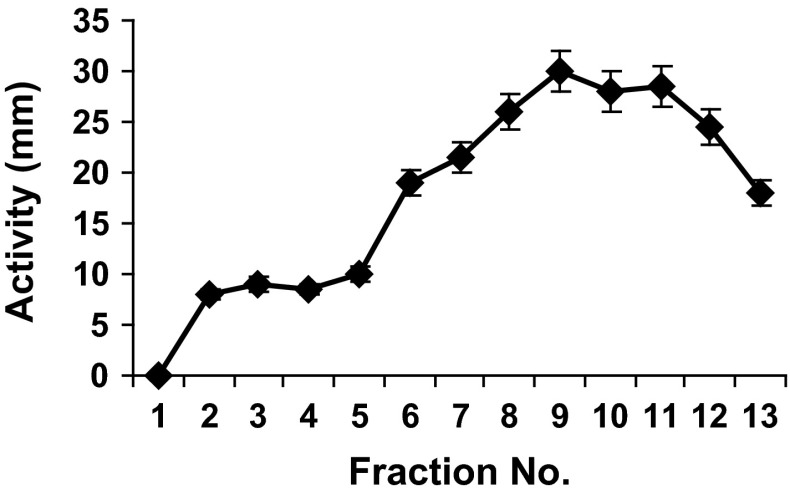
Elution pattern of peptide antibiotic from a Bio-Gel P-10 column equilibrated in 50-mM Tris buffer, pH 7.5. Fractions were collected and measured for activity (zone of inhibition)

The sample eluted from the column at two distinct peaks (Fig. 2). Initially high-molecular weight components were eluted which are above 6 kDa and not of our interest. Fractions corresponding to molecular weight of about 1.5 kDa were further purified using RP-HPLC. As the initial fractions also showed activity, these may represent the aggregated form of lipopeptide present in the sample.

### Reverse Phase HPLC

**Fig. 3:**
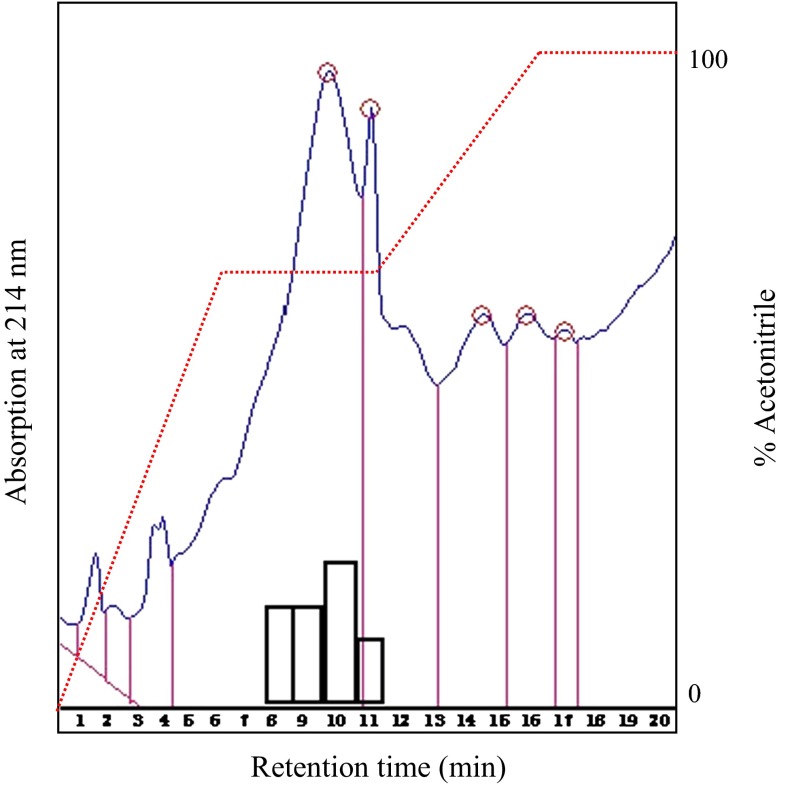
Purification of lipopeptides by reversed-phase HPLC on a Thermo Hypersil-Keystone ODS column. A segmented gradient was used and product eluted at 75 % of acetonitrile. Flow rate was maintained at 1 mL/min

Figure 3 shows the chromatogram of the HPLC purification of samples obtained from TLC, SPE, and gel filtration. Four active fractions were observed at 75 % acetonitrile with retention time ranging from 8 to 11 min. However, maximum activity was observed in fraction corresponding to retention time 10 min. This fraction was further analyzed through Tricin PAGE and MALDI–TOF-MS for their molecular mass determination.

### Tris-Tricine SD-PAGE

Tris-Tricine SDS was unable to discriminate between the lipopeptides produced by the strain BIA; however, it confirmed the purity of sample preparation. The purified product was considerably smaller than the marker band with lowest molecular weight and is free of any contaminating material as evidenced by the lack of any other bands. As a comparison, the culture supernatant produced many bands of stained contaminated material. A direct activity assay confirmed that the purified product is about 3 kDa, as revealed by the zone of inhibition on test organism plate.

### MALDI–TOF Mass Spectrometry

**Fig. 4:**
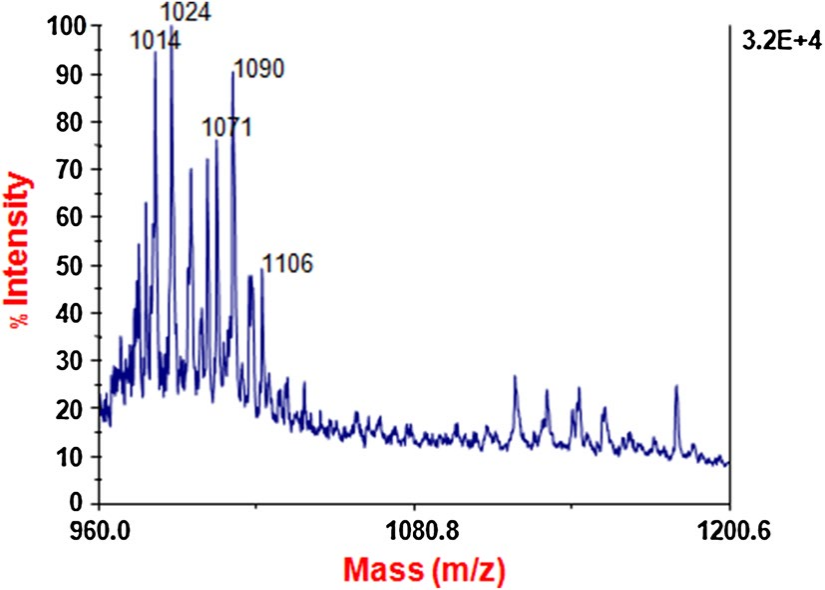
MALDI–TOF-MS analysis of surfactin and iturin like compounds produced by *B. subtilis* strain BIA

Purified product from RP-HPLC and whole bacterial cells were selected for analysis with MALDI–TOF-MS. When whole bacterial cells were used, these did not produce a good spectrum (data not shown). In the linear mode, we observed two clusters of peaks with different mass/charge (*m*/*z*) ratios for purified product (Fig. 4). One cluster was observed with *m*/*z* ratios between 1022 and 1036 which may be regarded as surfactin isoforms. The peak with a *m*/*z* ratio 1022.7 corresponds to the mass of [M + H]^+^ ion of surfactin with a fatty acid chain length of 14 carbon atoms. Other cluster was detected with mass/charge (*m*/*z*) ratios between 1070 and 1112.6, which could be attributed to protonated iturin isoforms. For example, the peak with a *m*/*z* of 1070.6 corresponds to the mass of [M + H]^+^ ion of iturin with a fatty acid chain length of 16 carbon atoms. Mass spectrometric analysis revealed that surfactin and iturin like compounds are produced by *B. subtilis* strain BIA.

## Discussion

Lipopeptides produced by *Bacillus subtilis* are members of a particular antibiotic class that included surfactin, iturin, and fengycin families [27]. These are non-ribosomally generated peptides and the synthesis is directed by large multienzyme complexes that have a modular structural organization and are thought to orderly link the amino-acid residues of the final peptide [28]. These represent potent biosurfactants and show efficient antimicrobial and antiviral properties, and, thus, are valuable, industrially important compounds. Studying the HPLC analysis of the culture supernatants of seven *Bacillus subtilis* strains, Ahimou et al. [29] showed that lipopeptide profile varied greatly according to the strain. Coproduction of surfactin and iturin or plipastatin B1, an inhibitor of phospholipase A_2_ [30], has also been reported in some *B. subtilis* strains [31–33]. This paper describes the extraction and purification of lipopeptide antibiotics from a newly isolated *B. subtilis* BIA strain. Table 1 outlines the effectiveness of each purification protocol. On the basis of these results, acid precipitation of the culture supernatant followed by SPE, ethyl acetate extraction followed by TLC, and ammonium sulfate precipitation followed by gel filtration actually increased antibiotic activity relative to supernatant. SPE proved to be the optimum method of isolation, as it accomplished 20 % recovery of the initial antibiotic activity and increases specific activity to 7.3 fold over that of original sample. Gel filtration indicated that lipopeptides were eluted from the column in two different forms, that is, as a monomer (MW ≤1200 Da) and as an aggregate (MW >6000 Da). Lipopeptides like surfactins have the ability to form micelle structures that form high-molecular weight aggregates. Mulligan and Gibbs [15] took advantage of this property in a one-step method to purify and concentrate surfactin from the culture supernatant by ultrafiltration. The ability of surfactant molecules to form high-molecular aggregates allows them to be retained by relatively high-molecular weight cut-off membranes. HPLC purification provided a highly purified sample for molecular mass determination with an increase of specific activity to 10.69 fold. As previously reported [34], using whole-cell MALDI–TOF, mass spectrometry cellular products can be detected which either are attached to the cell surface or are integrated into the outer cell membrane. In this way, information on the secondary metabolites produced by a microorganism can be obtained in minutes with high precision and sensitivity with no need to fractionate and purify the detected compounds. However, in this study, we were not able to produce a good spectrum from the whole-cell analysis. The purified product from RP-HPLC revealed that *B. subtilis* strain BIA produced a C_14_ surfactin and C_16_ iturin like compounds based on their molecular masses. The co-production by this strain is an interesting characteristic with potential practical applications. Further characterization of these lipopeptides would have relevance to minimize the use of synthetic fungicides and surfactants.

**Table 1:** Purification of lipopeptides from *B. subtilis* BIA

Sample	Sample volume (mL)	Total activity (AU)	Total absorbance (*A*_280 nm_)	Specific activity (AU/A_280_)	Activity recovered (%)	Fold purification
Supernatant	120	18,720	680	28	100	1
Solvent extraction	3	17,244	253	68	92	2.42
TLC	0.5	2808	19	148	15	5.29
HCl ppt.	5	15,724	171	92	84	3.28
SPE	1	3744	18	208	20	7.43
ASP	5	13,104	260	50	70	1.8
Gel filtration	1	2246	21	107	12	3.82
HPLC peak	1	1497	5	300	8	10.69
